# Persistent ^129^Xe MRI Pulmonary and CT Vascular Abnormalities in Symptomatic Individuals with Post-Acute COVID-19 Syndrome

**DOI:** 10.1148/radiol.220492

**Published:** 2022-06-28

**Authors:** Alexander M. Matheson, Marrissa J. McIntosh, Harkiran K. Kooner, Justin Lee, Vedanth Desaigoudar, Elianna Bier, Bastiaan Driehuys, Sarah Svenningsen, Giles E. Santyr, Miranda Kirby, Mitchell S. Albert, Yurii Shepelytskyi, Vira Grynko, Alexei Ouriadov, Mohamed Abdelrazek, Inderdeep Dhaliwal, J. Michael Nicholson, Grace Parraga

**Affiliations:** ^1^Robarts Research Institute; ^2^Department of Medical Biophysics; ^3^Department of Physiology and Pharmacology, Western University, London Canada; ^4^Center for In Vivo Microscopy, Duke University Medical Center, Durham NC USA; ^5^Division of Respirology, Department of Medicine, McMaster University, Hamilton Canada; ^6^Translational Medicine Program, Hospital for Sick Children, Toronto Canada; ^7^Department of Medical Biophysics, University of Toronto, Toronto Canada; ^8^Department of Physics, Ryerson University, Toronto Canada; ^9^Chemistry Department, Lakehead University; ^10^Thunder Bay Regional Health Research Institute; ^11^Northern Ontario School of Medicine, Thunder Bay Canada; ^12^Department of Physics and Astronomy; ^13^Department of Medical Imaging; ^14^Division of Respirology, Department of Medicine, Western University, London Canada.

## Abstract

**Background:**

In patients with post-acute COVID-19-syndrome (PACS), abnormal gas-transfer and pulmonary vascular density have been reported, but such findings have not been related to each other, or to symptoms and exercise limitation. The pathophysiological drivers of PACS in ever- and never-hospitalized patients are not well-understood.

**Purpose:**

To determine the relationship of persistent symptoms and exercise limitation with ^129^Xe MRI and CT pulmonary vascular measurements in individuals with PACS.

**Materials and Methods:**

In this prospective study, patients with PACS aged 18-80 years with a positive PCR COVID test were recruited from a quaternary-care COVID-19 clinic between April and October 2021. Participants with PACS underwent spirometry, diffusing-capacity-of-the-lung- for-carbon-monoxide (DLco), ^129^Xe MRI, and chest CT. Healthy controls had no prior history of COVID-19 underwent spirometry, DLco, and ^129^Xe MRI. The ^129^Xe MRI red-blood-cell (RBC) to alveolar-barrier signal ratio, RBC area-under-the-curve (AUC), CT volume-of-pulmonary-vessels with cross-sectional-area <5mm^2^ (BV5), and total-blood-volume (TBV) were quantified. St. George's Respiratory Questionnaire (SGRQ), International Physical Activity Questionnaire (IPAQ) and modified Borg Dyspnea Scale (mBDS) measured quality-of-life, exercise limitation and dyspnea. Differences between groups were compared using Welch's T-tests or Welch's ANOVA. Relationships were evaluated using Pearson (r) and Spearman (ρ) correlations.

**Results:**

Forty participants were evaluated including six controls (mean age, 35±15 years[standard deviation], 3 women) and 34 participants with PACS (mean age, 53±13 years[SD], 18 women), of which 22 were never-hospitalized. The ^129^Xe MRI RBC:barrier ratio was lower in ever- hospitalized participants (P=.04) compared to controls. BV5 correlated with RBC AUC (ρ=.44,P=.03). The ^129^Xe MRI RBC:barrier ratio was related to DLco (r=.57,P=.002) and FEV_1_ (ρ=.35,P=.03); RBC AUC was related to dyspnea (ρ=-.35,P=.04) and IPAQ score (ρ=.45,P=.02).

**Conclusion:**

^129^Xe MRI measurements were lower in ever- hospitalized participants with post- acute COVID-19-syndrome, 34±25 weeks post-infection compared to controls. ^129^Xe MRI measures were associated with CT pulmonary vascular density, DLco, exercise capacity, and dyspnea.

ClinicalTrials.gov: NCT04584671

See also the editorial by Wild and Collier.

## Graphical abstract

**Figure:**
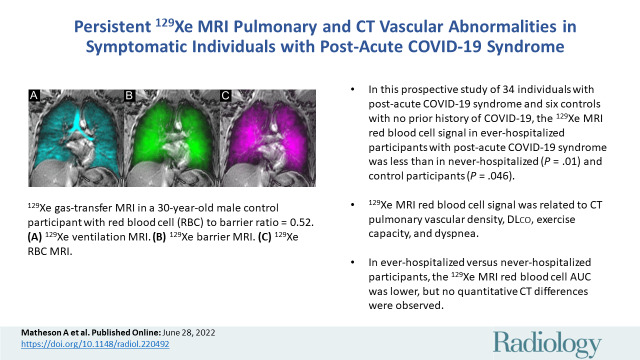

**Summary**

In symptomatic individuals with post-acute COVID-19 syndrome, ^129^Xe MRI gas exchange and CT vascular density measurements were abnormal and related to the diffusing capacity of the lung for carbon monoxide, the forced expiratory volume in 1 second, exercise limitation, and exertional dyspnea.

**Key Results**

■ In this prospective study of 34 individuals with post-acute COVID-19 syndrome and six controls with no prior history of COVID-19, the ^129^Xe MRI red-blood-cell signal in ever- hospitalized participants with post-acute-COVID-19-syndrome was less than in never- hospitalized (P=.01) and control participants (P=.046).
■ ^129^Xe MRI red-blood-cell signal was related to CT pulmonary vascular density, DLco, exercise capacity, and dyspnea.
■ In ever-hospitalized versus never-hospitalized participants, the ^129^Xe MRI red-blood-cell area-under-the-curve was lower, but no quantitative CT differences were observed.

## Introduction

The acute and post-acute phase of SARS-CoV-2 infection presents with a variety of symptoms,^1^ in patients who experienced mild infection^2^ and those hospitalized with more severe infection, requiring hospital-based care.^3^ The prevalence of post-acute COVID-19 symptomatic findings, including dyspnea at rest and on exertion, tachypnea, fatigue, exercise limitation, muscle weakness and cognition deficits, ranges from 20%^4^ to 81%.^3^ Such symptoms have been described with the umbrella term “post-acute COVID-19 syndrome” (PACS) defined as persistent symptoms or sequelae at least 12 weeks post-infection.^5^ Post-acute COVID symptoms are difficult to treat because the literature has reported varying degrees of abnormality in spirometry (FEV_1_ 2-20% below LLN)^6, 7^ and diffusing-capacity-of-the-lung for carbon-monoxide (22-88% below LLN)^6, 7^ alongside various CT abnormalities including ground glass opacities (41-89% present),^6, 7^ reticular patterns (0-67% present)^6, 7^ and atelectasis (33% present).^7^ A recent study showed that never-hospitalized patients also reported normal or nearly normal pulmonary function tests (6-37% abnormal at 4-month follow-up)^8^ and imaging was rarely available in these patients.

A recent CT pulmonary vascular investigation in hospitalized patients undergoing treatment has also suggested a shift in blood distribution from smaller to larger vessels,^9^ potentially due to microemboli and vascular remodelling affecting small-vessel resistance.^129^Xe gas-transfer MRI provides an opportunity to probe capillary-level abnormalities by detecting inhaled ^129^Xe dissolved in the alveolar membrane (quantified as barrier area-under-the-curve [AUC]) and red-blood-cells (quantified as RBC AUC). The ratio of ^129^Xe uptake (RBC:barrier ratio) has been observed to reflect impaired gas transfer in obstructive and restrictive disease^10^ and was also recently shown to detect low alveolar to red-blood-cell gas exchange in hospitalized COVID-19 patients 3-months post-discharge.^11,12^Although some long-term symptoms were reported in these patients, ^129^Xe MRI has not been performed in patients with PACS.

Most COVID-19 studies have been performed in ever-hospitalized patients,^3,12^ and report poor quality-of-life post-discharge. One recent study investigated symptoms post-infection in never- hospitalized patients.^2^ The most recent wave of COVID-19 infection has affected unprecedented numbers of people but with an apparently decreased rate of hospitalization due to less severe infection.^13^ Understanding the relationship between COVID-19 infectious severity and post- infection symptoms will be critical for health care planning as COVID-19 becomes endemic.

We hypothesized that long haul COVID-19 symptoms in the presence of normal pulmonary function would be associated with abnormal ^129^Xe MRI gas-exchange and CT pulmonary vascular density measurements and that such imaging measurements would differ in ever- and never- hospitalized PACS. Hence, in ever-COVID participants with PACS, we aimed to determine the relationship of persistent symptoms and exercise limitation to ^129^Xe MRI and CT pulmonary vascular measurements.

## Materials and Methods

### Study Participants

We prospectively evaluated individuals 18-80 years of age who provided written-informed consent to an ethics board (HSREB # 113224) Health Canada approved and registered protocol (ClinicalTrials.gov: NCT04584671). Study participants with a proven positive PCR COVID-19 test were prospectively recruited from a quaternary-care COVID-19 clinic between April and October 2021. Inclusion criteria consisted of: age ≥ 18 and <80 years, a documented case by positive RT_PCR test of COVID-19 infection that resulted in symptoms post-infection. Exclusion criteria consisted of: contraindications to MRI such as implants and severe claustrophobia, mental or legal incapacitation or could not read or understand written material, inability to perform spirometry or plethysmography maneuvers, and pregnancy. Healthy controls aged ≥ 18 and <80 years, with no prior history of COVID-19 or any other respiratory infection during the period February 2020- study visit date were recruited as a convenience sample in June 2021. Controls were excluded if there were clinically relevant incidental findings.

### Study Design

**Figure 1.**
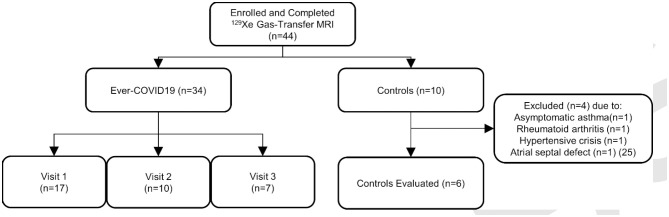
CONSORT flow diagram.

Figure 1 provides the study design which consisted of Visit 1 (3-months post +COVID test), an optional Visit 2 (9-months post +COVID test) and Visit 3 (15-months post +COVID test). Participants were administered salbutamol upon arrival at our centre and 15 minutes later performed post-bronchodilator (BD) spirometry and DLco immediately prior to MRI. Research thoracic CT was acquired within 30 minutes of MRI and then participants completed the six- minute-walk-test (6MWT) and Questionnaires (St. George's Respiratory Questionnaire (SGRQ),^14^ modified Medical Research Council (mMRC) Questionnaire, Chronic Obstructive Pulmonary Disease Assessment Test (CAT),^15^ post-COVID-19 Functional Status scale,^16^ International Physical Activity Questionnaire (IPAQ),^17^ modified Borg Dyspnea Scale (mBDS).^18,19^ ^129^Xe gas- exchange MRI was performed at either Visit 1, 2 or 3. SpO_2_ and heart rate were measured using an 8500 series handheld pulse oximeter (Nonin Medical Inc.) upon participant arrival as well as before and just after the 6MWT. For participants with PACS, the research visit was 35±25 weeks (range=6-79) post-COVID-19 infection with positive tests ranging from March 2020 to April 2021. Controls were evaluated in June 2021 after at least a single COVID-19 vaccine dose and none had experienced symptomatic respiratory illness for the period February 2020 up to and including the study visit date.

### Pulmonary Function Tests

Pulmonary function tests were performed according to American Thoracic Society guidelines^20,21^ using a *ndd EasyOne Pro LAB system* (ndd Medical Technologies). Post-BD measurements were performed 15 minutes after inhalation of 4×100 µg/inhalation salbutamol sulfate norflurane (Ivax Pharmaceuticals) using an *AeroChamber* (Trudell Medical International). Participants withheld inhaled medications before study visits according to American Thoracic Society guidelines (e.g. short-acting β-agonists ≥6 hours, long-acting β-agonists ≥12 hours, long-acting muscarinic antagonists ≥24 hours).^20^ Questionnaires and the 6MWT were self-administered under supervision of study personnel.

### ^129^Xe MRI

Anatomic ^1^H and ^129^Xe MRI were acquired using a 3.0 Tesla scanner (Discovery MR750; GE Healthcare) as previously described.^22^ ^129^Xe MRI were acquired using a flexible vest quadrature coil (Clinical MR Solutions). Gas-exchange ^129^Xe MRI and spectroscopy were performed, as described in Appendix E1,^22^ following coached inhalation and breath-hold of a 1.0L gas mixture (4/1 by volume ^4^He/^129^Xe for MRS, 1/1 by volume ^4^He ^129^Xe for MRI) from functional residual capacity. ^129^Xe magnetic resonance spectroscopy data were fit to three complex Lorentzian distributions to determine frequency and area under the curve (AUC). The RBC:barrier ratio was calculated as the ratio of RBC AUC to barrier AUC. Gas-transfer MRI data were reconstructed as described;^22^ additional detail is provided in Appendix E1.

### Thoracic CT

Within 30 minutes of MRI, CT was acquired post-BD after inhalation of 1.0L N_2_ from functional residual capacity, as previously described^22^ using a 64-slice LightSpeed VCT system (General Electric Healthcare; parameters: 64×0.625 collimation, 120 peak kilovoltage, 100 mA, tube rotation time=500ms, pitch=1.25, standard reconstruction kernel, slice thickness=1.25mm, field- of-view=40cm^2^), as previously described.^22^

Pulmonary vascular measurements included total blood volume (TBV), volume of pulmonary blood vessels ≤5mm^2^ (BV5), between 5-10mm^2^ (BV5-10) and >10mm^2^ (BV10), as detailed in Appendix E1. CT data were qualitatively evaluated by a single chest CT radiologist with >10 years’ experience (MA) for diagnostic and incidental findings. The qualitative reader was not blinded. CT data were also quantitatively evaluated by a single experienced observer (AMM) who was blinded to participant identification and clinical measurements using automated (Chest Imaging Platform, Brigham and Women's Hospital)^23^ software.

Data generated or analyzed during the study are available from the corresponding author by request and will be deposited at https://apilab.ca/our_code.html under data version 20220401.

### Statistical Analysis

The ^129^Xe MRI signal intensity ratio of RBC to alveolar tissue barrier was the primary endpoint. SPSS (SPSS Statistics 27.0; IBM) was used for all statistical analyses. Data were tested for normality using Shapiro-Wilk tests and nonparametric tests were performed for non-normally distributed data. Relationships were evaluated using Pearson (r) and Spearman (ρ) correlations. Intergroup differences were tested using Welch's t-tests for two-group or Welch's ANOVA for multi-group analyses. Fischer's exact tests were used for categorical variables. Results were considered statistically significant when the probability of making a type I error was <5% (p<0.05).

## Results

### Participant Characteristics

As shown in Figure 1, of an initial 44 participants, data were acquired in 34 participants with PACS (mean age, 53 years ±13[SD], 18 women) and 10 control participants (mean age, 35 years ±15[SD], five men), of which four controls were excluded due to clinically relevant incidental findings. Three control participants were excluded due to asymptomatic asthma, rheumatoid arthritis, and hypertensive crisis. Another participant was excluded due to an incidental finding that lead to the diagnosis of a large, asymptomatic atrial septal defect.^24^

For participants with PACS, the research visit was 35±25 weeks (range=6-79) post-COVID-19 infection with positive tests ranging from March 2020 to April 2021. Never-COVID participants were evaluated in June 2021 after at least a single COVID-19 vaccine dose and none had experienced symptomatic respiratory illness for the period up to and including February 2020. Table 1 summarizes participant demographic data for never- and ever-participants with PACS, as well as never- and ever-hospitalized participants with PACS. Control participants were younger (P=.02) than participants with PACS (controls 35±15 years, PACS 53±13 years) and had a lower BMI (controls 25±3 kg/m^2^, PACS 30±5 kg/m^2^; P=.02). Persistent symptoms that led to a diagnosis of PACS and follow-up by the London Health Sciences COVID clinic are summarized in Table E1. Most participants reported respiratory symptoms including exertional dyspnea as well as fatigue and brain fog. Among the ever-hospitalized COVID patients, two were treated in ICU and none required ventilation. Participant medications are summarized in Table E2.

**Table 1.**
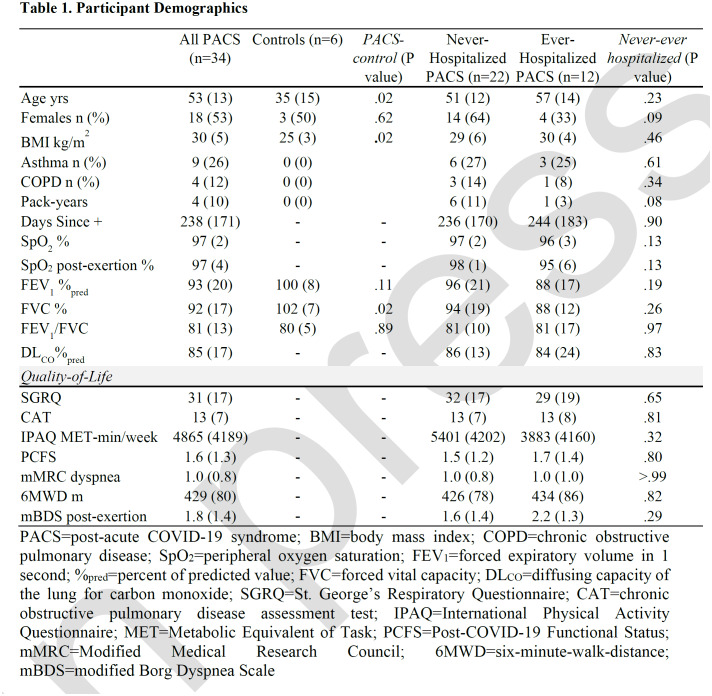
Participant Demographics

### Qualitative MRI and CT Findings

**Figure 2.**
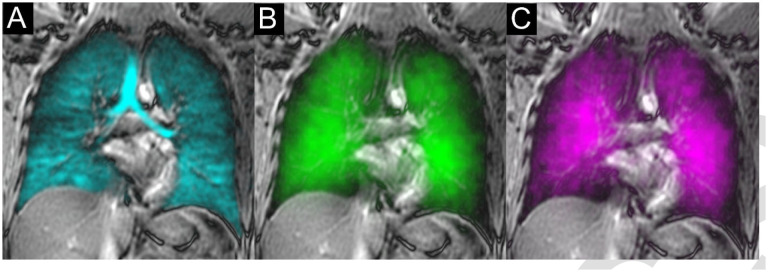
^129^Xe gas-transfer MRI in a 30-year-old male control participant with RBC:barrier ratio=0.52. A) ^129^Xe ventilation MRI B) ^129^Xe barrier MRI C) ^129^Xe red-blood-cell (RBC) MRI.

**Figure E2.**
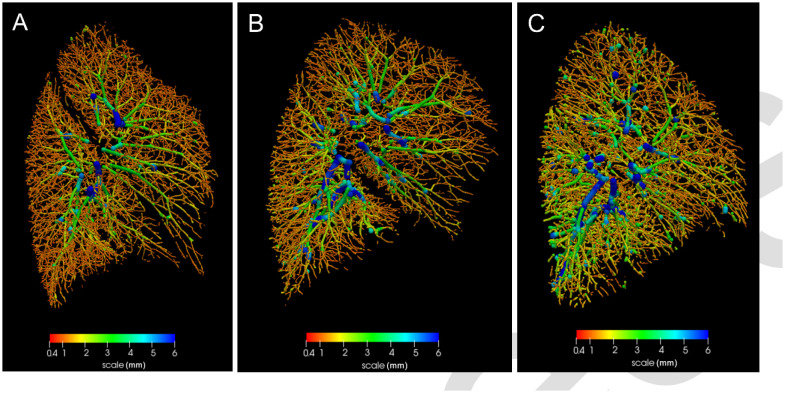
Evidence of pulmonary vascular abnormalities in participants with PACS. Abnormal BV5/TBV was associated with greater vessel caliber as measured by CT without a change in TBV. **(A)** a 59-year-old never-hospitalized female with PACS, BV5/TBV=62%. **(B)** a 69-year-old never-hospitalized male with PACS, BV5/TBV=45%. **(C)** A 70-year-old ever- hospitalized male with pre-existing COPD and PACS, BV5/TBB=35%.

Figure 2 shows representative ^129^Xe MRI ventilation, alveolar-capillary tissue barrier and RBC maps and thoracic CT in a never-COVID-19 participant, a never-hospitalized and an ever- hospitalized PACS participant. In the never-COVID control participant, there were homogeneous signal intensities for ventilation, alveolar-capillary tissue barrier and RBC compartments. In the never- and ever-hospitalized participants with PACS, there were patchy alveolar-capillary tissue barrier and RBC signal intensity maps. As shown in Figure E2C, in some participants with abnormal CT BV5/TBV, there was visual evidence of fewer small vessels and a greater density of larger vessels without a visually obvious change in TBV. A summary of CT radiological findings is included in Table E3. In never-hospitalized participants the most common findings were nodules (8/22, 36%), bronchiectasis (3/22, 14%), ground glass opacity (4/22, 18%) and atelectasis (3/22, 14%). In ever-hospitalized participants the CT findings were similar but with greater frequencies for ground glass opacity (5/12 42%) and consolidation (2/12, 17%).

### Differences Between Never- and Ever-hospitalized Participants

**Figure 3.**
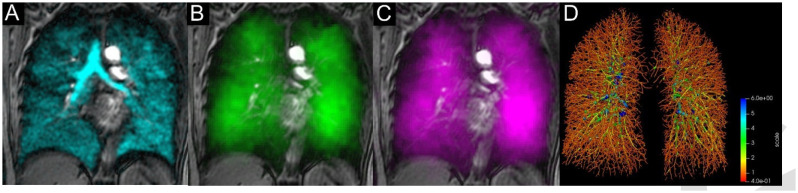
^129^Xe gas-transfer MRI and CT pulmonary vessels in a 59-year-old never-hospitalized female participant with PACS (RBC:barrier=0.26 and CT blood volume in vessels with cross- sectional area ≤5mm^2^ normalized to total blood volume (BV5/TBV)= 62%). A) ^129^Xe ventilation MRI B) ^129^Xe barrier MRI C) ^129^Xe red-blood-cell (RBC) MRI.

Table 2 shows the MRI (n=34) and CT pulmonary vascular measurements (n=24) by hospitalization status and Figure 3 shows some of these measurements in box and whisker plots. Five CT segmentations were excluded from the evaluation because of segmentation artifacts in regions of CT consolidation/opacities in ever-hospitalized participants.

**Table 2.**
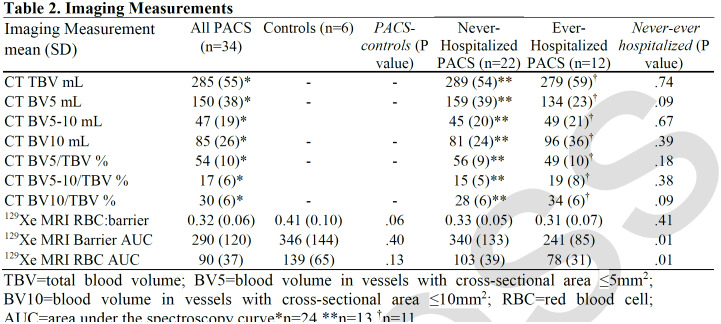
Imaging Measurements

As shown in Table 2, in all Participants with PACS as compared with controls participants, ^129^Xe MRI RBC:barrier ratio (0.32±0.06 vs. 0.41±0.10 *P*=.06) trended toward a difference. The ^129^Xe MRI barrier AUC (340±133 vs. 241±85, P=.01) and RBC AUC (103±39 vs. 78±31, P=.01) measures were greater in never- as compared with ever-hospitalized participants. There was no difference in BV5/TBV for never- (56±9) and ever-hospitalized participants (49±10; P=.14) although the trend observed was consistent with previous reports of vascular pruning in COVID-19.^9^ Figure 3 shows differences in box and whisker plots by participant group for ^129^Xe MRI RBC:barrier ratio, RBC and barrier AUC. Differences in RBC AUC were observed between never- COVID, never-hospitalized PACS and ever-hospitalized PACS.

Figure 5 shows proposed mechanisms underlying abnormal CT and MRI measurements.

**Figure 4.**
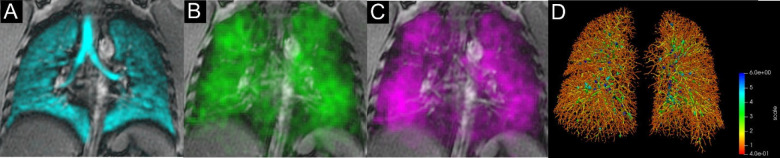
^129^Xe gas-transfer MRI and CT pulmonary vessels in a 42-year-old ever-hospitalized male participant with PACS (RBC:barrier=0.33 and CT blood volume in vessels with cross- sectional area ≤5mm^2^ normalized to total blood volume (BV5/TBV)=54%). A) ^129^Xe ventilation MRI B) ^129^Xe barrier MRI C) ^129^Xe red-blood-cell (RBC) MRI.

**Figure 5.**
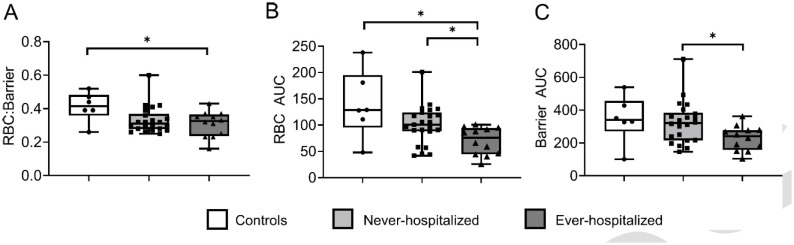
^129^Xe spectroscopy measurements for controls, never-hospitalized and ever- hospitalized participants with PACS. Controls and never-hospitalized participants with PACS reported different ^129^Xe MR spectroscopy measurements. **(A)** red-blood-cell to barrier ratio (RBC:barrier): controls (0.41±0.10) and ever-hospitalized PACS (0.31±0.07), P=.04. **(B)** RBC area-under-the-curve (AUC): controls (139±65) and ever-hospitalized PACS (78±31), P =.046, never-hospitalized PACS (103±39) and ever-hospitalized PACS, P=.01. **(C)** Barrier AUC: Never-hospitalized PACS (340±133) and ever-hospitalized PACS (241±85), P=.01

**Figure 6.**
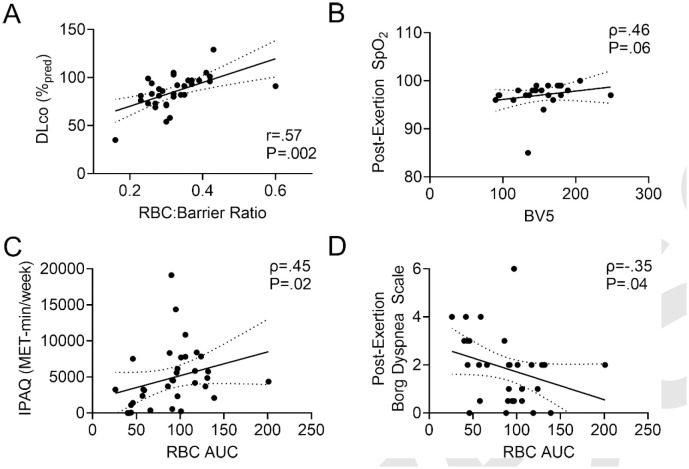
^129^Xe MR Spectroscopy measurement relationships with pulmonary function and exercise measurements in participants with PACS. **(A)**^129^Xe gas-transfer red-blood-cell to barrier ratio (RBC:barrier) measurements were related to (r=.57, Holm-Bonferonni P=.002) diffusing-capacity-of-the-lung for carbon monoxide (DLCO). **(B)**^129^Xe MRI RBC area-under-the-curve (AUC) trended towards an association with CT blood volume in vessels with cross-sectional area ≤5mm^2^ (BV5; ρ=.46, Holm-Bonferonni P=.06). **(C)**^129^Xe MR RBC AUC was related to International Physical Activity Questionnaire (IPAQ) exercise capacity (ρ=.45, Holm-Bonferonni P=.02). **(D)**^129^Xe MR RBC AUC was related to dyspnea measured by post-exertion modified Borg Dyspnea Scale (ρ=-.35, Holm-Bonferonni P=.04).

**Figure 7.**
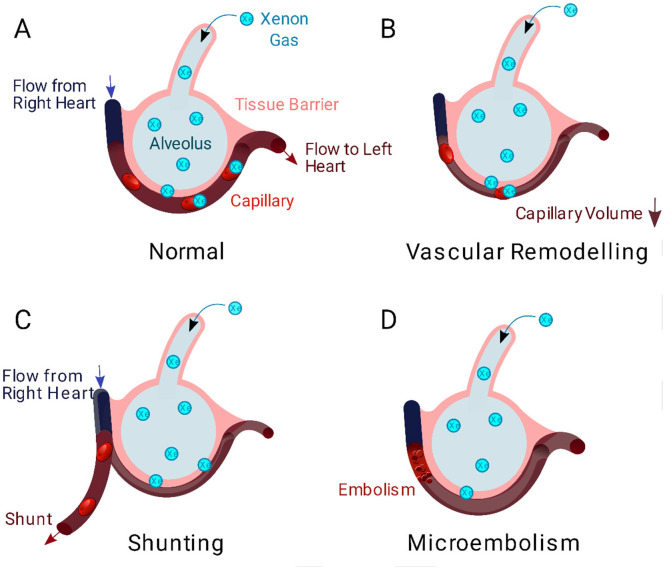
Proposed Mechanisms explaining relationships for ^129^Xe MRI RBC AUC **(A)** Gas-exchange in a healthy individual occurs as xenon diffuses through the tissue barrier and attaches to RBC. **(B)** Vasoconstrictive remodeling following infection reduces the available blood volume for ^129^Xe binding. **(C)** Changes to vascular resistance and flow patterns may result in redistributions of pulmonary blood through shunting away from ^129^Xe ventilated regions. **(D)**Thrombus or microembolism blocks capillary-level bloodflow, preventing ^129^Xe uptake in RBC and redistributing blood upstream in the vasculature.

### Relationships between imaging measurements, symptoms, and exercise limitation

Figure 4 shows the relationships for CT and MRI measurements with one another and with symptoms and exercise limitation. Table E4 shows all MRI and CT relationships with spirometry, DLCO, questionnaire and exercise data. Figure 4 shows that the ^129^Xe MRI RBC:barrier ratio was correlated with DLCO (r=.57, P=.002) and FEV_1_ (ρ=35, P=.03). The ^129^Xe MRI RBC AUC was correlated with CT BV5 (ρ=.44, P=.03), IPAQ score (ρ=.45, P=.02), post-exertional SpO_2_ (ρ=.37, P=.03) and post-6MWT Borg breathlessness (ρ=-.35, P=.04), but not SGRQ score (r=-.15, P=.40). BV5 was also correlated with post-exertional SpO_2_ (ρ=.46, P=.03).

Table E4 provides additional relationship data without statistical tests.

## Discussion

Independent studies^11,12^ have uncovered evidence of either MRI or CT pulmonary vascular abnormalities in previously hospitalized patients with COVID-19 who were recovered from infection but remained symptomatic. Here we endeavored to determine if ^129^Xe MRI abnormalities were present in never-hospitalized Participants with PACS and to determine relationships between MRI and CT measurements with clinical and patient-centred measurements. We evaluated 40 participants, including 22 never-hospitalized and 12 ever-hospitalized participants with PACS, 35 ±25 weeks post COVID infection and observed: 1) different ^129^Xe MRI RBC:barrier ratio (0.31±0.07 vs 0.41±0.10; P=.04) and RBC AUC (90±37 vs 139±65; P=.046) in ever- hospitalized participants with PACS with normal spirometry (but abnormal SGRQ, IPAQ, mMRC) as compared with controls, 2) differences in ever- as compared with never-hospitalized participants ^129^Xe MRI RBC (78±31 vs 103±39; P=.01) and barrier AUC (241±85 vs 340±133; P=.01), 3) relationships for MRI RBC:barrier ratio with DLCO (r=.57, P=.002) and FEV_1_ (ρ=.35, P=.03), and, 4) relationships for MRI RBC AUC with CT BV5(ρ=.44, P=.03), IPAQ score (ρ=.45, P=.02), post-exertional SpO_2_ (ρ=.46, P=.03) and post-6MWT dyspnea (ρ=-.35, P=.04).

In all patients with COVID-19, mean spirometry values were normal and mean DLco was at the bottom of the normal range and SGRQ, IPAQ, and mMRC scores were abnormal. In addition, RBC:barrier ratio (controls 0.41±0.10, participants with ever-COVID 0.32±0.06; P=.06) trended toward a difference as compared with never-COVID controls. As in previous studies,^25^ we used hospitalization status to dichotomize post-COVID patients and detected MRI differences in never- and ever-hospitalized participants including ^129^Xe MRI RBC and barrier AUC. Whereas the CT measurements in never-hospitalized participants were similar to never-COVID values previously reported (BV5/TBV=56%, BV10/TBV=28%),^9^ CT pulmonary vascular measurements in ever- hospitalized COVID-19 patients were consistent with vascular pruning, similar to previous findings.^9^ CT evidence of “vascular pruning” has been hypothesized to be due to vasoconstrictive remodelling of the capillary systems and small blood vessels.^9^ While the capillaries are well beyond the spatial resolution of CT, histologic analyses^26^ have indicated that capillary remodelling occured in COPD patients when CT vascular pruning was also identified. In never-hospitalized patients, we observed ^129^Xe MRI, but not CT abnormalities. Since MRI directly probes the function of the alveolar-capillary boundary, it may be more sensitive or more targeted than CT to microvascular abnormalities.

Together, the abnormal MRI and CT findings were consistent with abnormal gas exchange stemming from the alveolar tissue barrier and pulmonary vascular compartments. Similar to previous reports of post-COVID coagulation and emboli,^1^ it is possible that we were measuring micro-embolic or micro-thrombotic obstruction of small capillaries which explained the abnormal RBC signal. Other vascular changes, such as vascular injury, vascular remodelling or shunting may also be possible and has previously been hypothesized post-COVID-19.^5,9,25^ Post-mortem micro-CT imaging of COVID-19 infection supports these interpretations as abnormal alveolar- level structures and occluded capillaries were observed.^27^

We observed relationships for ^129^Xe MRI RBC:barrier ratio with DLco, FEV_1_. Whilst modestly low DLco is common in PACS patients, post-COVID hospitalization,^28^ a pilot ^129^Xe MRI study unexpectedly did not find a DLco and MRI gas-transfer relationship.^12^ In contrast, here we observed relationships for the ^129^Xe MRI RBC:barrier ratio with DLco and FEV_1_. The relationship with DLco was not unexpected because previous work showed these relationships in both obstructive and restrictive lung disease.^29^ RBC:Barrier and FEV_1_ relationships have not previously been observed but could reflect underlying tissue changes in participants that also impact airway restriction. In our study, DLco was greater than 80%pred in both never- and ever-hospitalized participants and FEV_1_ was also normal which together may suggest that the ^129^Xe MRI RBC:barrier ratio is highly sensitive to pulmonary gas-transfer abnormalities.

We also observed a moderate correlation between BV5 and RBC AUC. This finding supports a link between RBC gas uptake and small-vessel abnormalities in PACS. Microvascular remodelling, shunting, thrombuses, micro-embolisms, or some combination of these may play a role. Increased vascular resistance due to these structural modifications could also explain how such abnormalities are also visible throughout the vascular tree. Hemodynamic measurements were outside the scope of our study but may prove an important subject of future investigation into PACS mechanisms.

We were surprised to detect relationships for MRI RBC AUC with post-exertion SpO_2_, exertional dyspnea (modified Borg Dyspnea Scale) and IPAQ score. Similar to previous studies of post- COVID patients,^5,25,30^ in our study, there was abnormal SGRQ (31±17 vs. 6±9 in general population^31^), CAT (13±7, >90^th^ percentile general population^32^) and mMRC dyspnea (1.0±0.7, >91^st^ percentile general population^33^). Whilst there were no relationships for MRI and CT measurements with SGRQ (which is validated for use in COPD),^14^ there was a correlation for IPAQ activity and MRI RBC AUC. Relationships between MRI, CT, pulmonary function and symptoms suggest a physiologic mechanistic link. Abnormal gas transfer, demonstrated by the relationship between RBC:barrier and DLco, would lead to poor oxygenation and vascular changes, possibly reflected in the trend towards a relationship between post-exertion SpO_2_ and CT BV5. Vascular abnormality-driven desaturation could explain commonly reported symptoms in PACS such as exercise limitation and dyspnea,^3^ which we observed to be related to RBC AUC. Pulmonary vascular abnormalities including the low RBC signal (which is a surrogate for abnormal O_2_ uptake) may stem from vascular remodeling, where narrowed vessels reduce the available blood volume, or eliminated altogether in regions with vascular shunting or persistent microemboli. For example, in cadaveric COVID lungs, there was histological evidence of severe endothelial damage and distorted, elongated vessels alongside microemboli.^34^ Shunting has been observed during infection in patients with COVID^35^ and perfusion of damaged or unventilated alveoli also would also reduce RBC signal in the lung. These potential mechanisms are supported by the relationship between the MRI RBC:barrier ratio and DLco, and an RBC AUC relationship with SpO_2_. Microvascular changes in flow and resistance could have upstream effects on the vasculature and may explain blood redistribution observed here and in other studies.^9^ The relationship between RBC AUC, dyspnea scores and exercise capacity measured by IPAQ help explain dyspnea and exercise impairment in some post-COVID patients as pulmonary vascular gas-exchange dysfunction.

In our study, the range of follow-up was quite wide (6-79) weeks post-positive test) with most COVID-19 testing at our centre performed approximately 1-week post-infection. While post-acute infectious symptoms were potentially possible, the emerging literature now describes the timelines for clinically relevant post-covid symptoms that include 4-6 weeks post-infection. For example, The Centers for Disease Control and Prevention (CDC) coined the term post-COVID condition as “a wide range of new, returning, or ongoing health problems people can experience four or more weeks after first being infected with the virus that causes COVID-19”^36^. The World Health Organization (WHO) also describes the post-COVID-19 condition, typically three months from the onset of COVID-19.^37^ As an alternative that blends both consensus definitions, The National Institute for Health and Care Excellence (NICE)^38^, coined the term long-COVID as signs and symptoms that continue or develop following the acute infectious phase of COVID-19, which includes both ongoing symptomatic COVID-19 and post-COVID-19 syndrome all greater than 4 weeks post infection.^5^ Hence our understanding and these definitions are still quite fluid. Given these definitions, the ever-COVID participants evaluated in our study can be considered as having post-acute COVID-19 syndrome or long COVID, based on their symptoms and timeframe since symptomatic infection.

We recognize a number of study limitations. For example, the relatively small sample size of the control and PACS subgroups, certainly limits the generalizability of our findings. Our study was not powered based on ^129^Xe MRI spectroscopy measurements so our results must be considered exploratory and hypothesis generating. To provide a transparent snapshot of our results with the COVID-19 research community, we provided data in Appendix E1 without statistical tests so that other centres may utilize our results to help generate sample sizes for long term follow-up studies. Other limitations include: 1) CT was not acquired in the control subgroup which prevented CT comparisons across all three subgroups; 2) all participants were referred from a COVID-19 clinic focusing on long-haul symptoms and therefore recruitment was likely biased towards symptomatic individuals seeking some form of explanation or intervention; 3) participants with PACS were older than the controls (53±13 years vs 35±15). To our knowledge, the effect of age on ^129^Xe gas- exchange biomarkers has not been reported. However, it is possible that similar to age-related changes observed for DLco,^39^ age may also influence MRI gas-transfer measurements; 4) COVID- 19 antibody testing was not performed to verify COVID-infection status in the never-Covid volunteers, so while unlikely, it is possible that some may have previously experienced an asymptomatic infection prior to the study; 5) the mean RBC:barrier ratio estimated for the control subgroup was lower than previous reports^11,12^ and this means that the differences detected for COVID patients may be conservative underestimates; 6) ^129^Xe gas-exchange MRI was performed on one of Visit 1, 2 or 3 which broadened the time post-COVID infection to 35±25 weeks. As shown in Appendix E1, there was no bias over time towards improved gas-exchange but nevertheless, it will be important to evaluate those participants who performed MRI at Visit 1 for potential longitudinal differences; and finally, 7) MR image heterogeneity was not evaluated quantitatively in our study and we note that previous ^129^Xe MRI COVID-19 investigations^11,12^ also reported the RBC:barrier ratio which makes comparisons with our study possible. Unfortunately, gas-exchange imaging was not technically implemented at our centre until our COVID-19 study was already underway for a year and in these participants, MR spectroscopy was implemented first for our study.

Larger studies aimed at identifying mechanistic relationships between dyspnea and other symptoms with ^129^Xe MRI abnormalities are complex to undertake in participants with PACS. The findings of abnormal ^129^Xe MRI gas-exchange measurements in never-hospitalized COVID patients and the relationships between ^129^Xe MRI and CT pulmonary vascular measurements have not been previously established in the literature. In our study, both CT and ^129^Xe MRI suggest temporally persistent pulmonary vascular density and gas-transfer abnormalities that were related to exercise limitation and exertional dyspnea. We observed abnormal ^129^Xe MRI gas-exchange measurements in never-hospitalized participants with COVID and some ^129^Xe MRI measurements were worse in ever-hospitalized patients compared to controls. We also detected relationships between ^129^Xe MRI and CT pulmonary vascular measurements that point to persisting pulmonary vascular abnormalities including vessel density and gas-transfer abnormalities that were related to exercise limitation and exertional dyspnea. Furthermore, future studies will seek to determine if pulmonary vascular abnormalities can act as a predictor of long-term PACS outcomes and if abnormal gas-exchange measures can predict recovery. Pulmonary vascular pathologies play a role in PACS regardless of COVID-19 severity.

## Figures and Tables

**Table E1.**
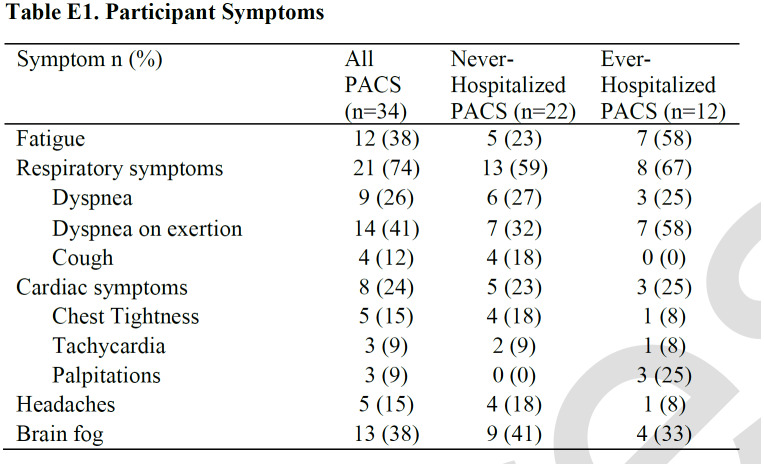
Participant Symptoms

**Table E2.**
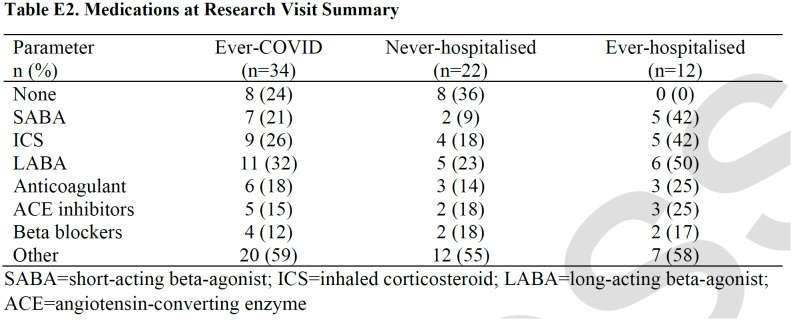
Medications at Research Visit Summary

**Table E3.**
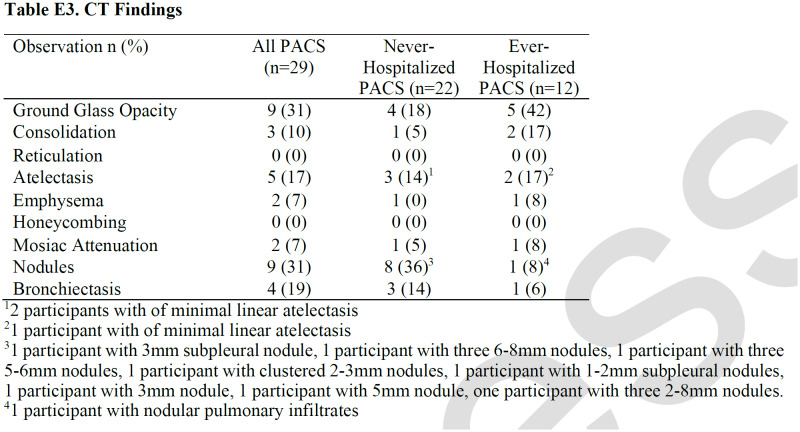
CT Findings

**Table E4.**
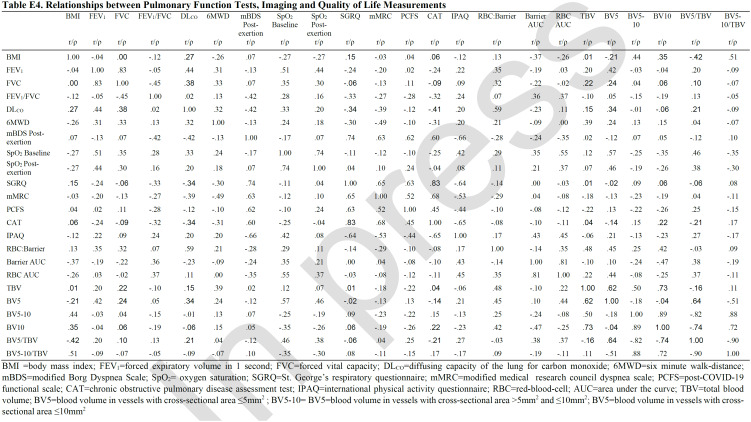
Relationships between Pulmonary Function Tests, Imaging and Quality of Life Measurements

**Figure E1.**
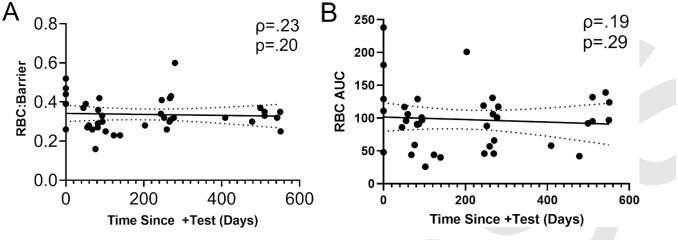
**(A)** Gas-exchange measurements in never-COVID and PACS participants at varying imaging dates post-infection. Never-COVID participant data were denoted as zero days since positive test. RBC:barrier ratio was not significantly different over time (ρ=.23, P=.20). **(B)** Gas- exchange measurements in never-COVID and PACS participants at varying imaging dates post- infection. Never-COVID participant data were denoted as zero days since positive test. RBC AUC was not significantly different over time (ρ=.19, p=.29)

## Appendix E1

### Methods

Table E1 summarizes symptoms reported in PACS participants in this study.

Anatomic ^1^H MRI was acquired using a fast-spoiled gradient-recalled-echo sequence (partial-echo acquisition; total acquisition time, 8 seconds; repetition-time msec/echo time msec, 4.7/1.2; flip- angle, 30°; field-of-view, 40×40cm^2^; bandwidth, 24.4 kHz; 128×80 matrix, zero-filled to 128×128; partial-echo percent, 62.5%; 15-17×15mm slices). ^129^Xe MR spectroscopy was acquired following inhalation breath-hold of a 1.0L gas mixture (4/1 by volume 4He/129Xe) from functional residual capacity (FRC) using a free-induction-decay whole-lung spectroscopy sequence (200 dissolved- phase spectra, TR=15ms, TE=0.7ms, flip=40°, BW=31.25kHz, 600μs 3-lobe Shinnar-Le Roux pulse). Spectroscopy was used to determine the echo time for a 90° barrier/RBC phase difference (TE90). ^129^Xe MRI was performed following inhalation of a 1.0L gas mixture (1/1 by volume ^4^He/^129^Xe) using an interleaved gas/dissolved-phase 3D radial sequence (TR=15ms TE=variable, flip=0.5°/40°, FOV=40cm^3^, matrix=72×72×72, BW=62.5kHz, 990 gas/dissolved projections, 600μs 3-lobe Shinnar-Le Roux pulse, frequency shift=7.664kHz). Supine participants were coached to inhale a 1.0L bag (Tedlar; Jensen Inert Products, Coral Springs, FL, USA) (500mL ^129^Xe + 500mL ^4^He for ^129^Xe MRI and 1.0L N2 for ^1^H MRI) from the bottom of a tidal breath (functional residual capacity) with acquisition under breath-hold conditions. ^129^Xe gas was polarized to 30-40% (Polarean; Xenispin 9820, Durham, NC, USA).^1^

Gas-transfer MRI data were reconstructed as previously described using a re-gridding method for non-cartesian acquisition.^2^ Receiver phase-offset and local phase inhomogeneity were corrected as previously described.^3^

^129^Xe gas-exchange MRI were corrected for local phase inhomogeneity using acquired interleaved gas-compartment data. Deviations from uniform phase in the gas image were assumed to result from phase inhomogeneity and voxel-wise phase corrections were applied to eliminate inhomogeneity effects. Receiver phase-offset was corrected using the spectroscopic RBC:barrier ratio. A phase correction Δ ϕ was applied such that the ratio of real to imaginary channel signal matched the spectroscopic RBC:barrier ratio under the assumption that RBC and barrier signal should be perfectly aligned to the real and imaginary channels, respectively, at TE_90_.

Within 30 minutes of MRI, CT was acquired post-BD after inhalation of 1.0L N_2_ from functional residual capacity using a 64-slice LightSpeed VCT system (General Electric Healthcare, Milwaukee, WI, USA; parameters: 64×0.625 collimation, 120 peak kilovoltage, 100 mA, tube rotation time=500ms, pitch=1.25, standard reconstruction kernel, slice thickness=1.25mm, field- of-view=40cm^2^) as previously described ^4^. The total-effective-dose (1.8 mSv) was calculated using the ImPACT patient dosimetry calculator (UK Health Protection Agency NRPB-SR250 software). CT vessel measurements were performed in Chest Imaging Platform using a fully-automated pipeline. Images were filtered with a median filter before being passed to a thresholding script to provide a basic segmentation of left and right lungs for region-of-interest identification. Next, a scale-space particle system^5^ was used to identify vessels within the region-of-interest by computing multi-scalar maps of local Hessian features, i.e. calculating how tube-like local regions appear. An optimization moves sampling particles to tube-like regions of the image, where particle properties represent geometric properties of the underlying structure (radius/scale, shape, orientation). A kernel-density approach was used to compute a distribution of vessel-volumes at varying vessel cross-sections. This distribution was used to calculate BV5, BV5-10 and BV10. Vessel visualization was performed in Paraview (Kitware Inc., New York, NY, USA).

## References

[1] Nurek M , Rayner C , Freyer A , Taylor S , Jarte L , MacDermott N , et al . Recommendations for the recognition, diagnosis, and management of long COVID: a Delphi study . Br J Gen Pract . 2021 ; 71 ( 712 ): e815 – e25 . 34607799 10.3399/BJGP.2021.0265 PMC8510689

[2] Estiri H , Strasser ZH , Brat GA , Semenov YR , Patel CJ , Murphy SN , et al . Evolving phenotypes of non-hospitalized patients that indicate long COVID . Bmc Med . 2021 ; 50 ( 3 ) . 10.1186/s12916-021-02115-0 PMC8474909 34565368

[3] Fernandez-de-las-Penas C , Palacios-Cena D , Gomez-Mayordomo V , Rodriuez-Jimenez J , Palacios-Cena M , Velasco-Arribas M , et al . Long-term post-COVID symptoms and associated risk factors in previously hospitalized patients: A multicenter study . J Infection . 2021 ; 83 ( 2 ): 271 – 4 . 10.1016/j.jinf.2021.04.036 PMC8110627 33984399

[4] Sandmann FG , Tessier E , Lacy J , Kall M , Van Leeuwen E , Charlett A , et al . Long-term health-related quality of life in non-hospitalised COVID-19 cases with confirmed SARS-CoV-2 infection in England: Longitudinal analysis and cross-sectional comparison with controls . Clin Infect Dis . 2022 . 10.1093/cid/ciac151 PMC8903473 35245941

[5] Nalbandian A , Sehgal K , Gupta A , Madhavan MV , McGroder C , Stevens JS , et al . Post- acute COVID-19 syndrome . Nature Medicine . 2021 ; 27 ( 4 ): 601 – 15 . 10.1038/s41591-021-01283-z PMC8893149 33753937

[6] Huang C , Huang L , Wang Y , Li X , Ren L , Gu X , et al . 6-month consequences of COVID- 19 in patients discharged from hospital: a cohort study . Lancet . 2021 ; 397 ( 10270 ): 220 – 32 . 33428867 10.1016/S0140-6736(20)32656-8 PMC7833295

[7] van Gassel RJJ , Bels JLM , Raafs A , van Bussel BCT , van de Poll MCG , Simons SO , et al . High Prevalence of Pulmonary Sequelae at 3 Months after Hospital Discharge in Mechanically Ventilated Survivors of COVID-19 . American Journal of Respiratory and Critical Care Medicine . 2021 ; 203 ( 3 ): 371 – 4 . 33326353 10.1164/rccm.202010-3823LE PMC7874313

[8] Munker D , Veit T , Barton J , Mertsch P , Mummler C , Osterman A , et al . Pulmonary function impairment of asymptomatic and persistently symptomatic patients 4 months after COVID-19 according to disease severity . Infection . 2022 ; 50 ( 1 ): 157 – 68 . 34322859 10.1007/s15010-021-01669-8 PMC8318328

[9] Lins M , Vandevenne J , Thillai M , Lavon BR , Lanclus M , Bonte S , et al . Assessment of Small Pulmonary Blood Vessels in COVID-19 Patients Using HRCT . Acad Radiol . 2020 ; 27 ( 10 ): 1449 – 55 . 32741657 10.1016/j.acra.2020.07.019 PMC7381940

[10] Wang Z , Bier EA , Swaminathan A , Parikh K , Nouls J , He M , et al . Diverse cardiopulmonary diseases are associated with distinct xenon magnetic resonance imaging signatures . Eur Respir J . 2019 ; 50 ( 3 ) . 10.1183/13993003.00831-2019 PMC7271066 31619473

[11] Li H , Zhao X , Wang Y , Lou X , Chen S , Deng H , et al . Damaged lung gas exchange function of discharged COVID-19 patients detected by hyperpolarized (129)Xe MRI . Sci Adv . 2021 ; 50 ( 3 ) . 10.1126/sciadv.abc8180 PMC7775756 33219111

[12] Grist JT , Chen M , Collier GJ , Raman B , Abueid G , McIntyre A , et al . Hyperpolarized (129)Xe MRI Abnormalities in Dyspneic Patients 3 Months after COVID-19 Pneumonia: Preliminary Results . Radiology . 2021 ; 301 ( 1 ): E353 – E60 . 34032513 10.1148/radiol.2021210033 PMC8168952

[13] Maslo C , Friedland R , Toubkin M , Laubscher A , Akaloo T , Kama B. Characteristics and Outcomes of Hospitalized Patients in South Africa During the COVID-19 Omicron Wave Compared With Previous Waves . JAMA . 2021 . 10.1001/jama.2021.24868 PMC8719272 34967859

[14] Jones PW , Quirk FH , Baveystock CM , Littlejohns P. A self-complete measure of health status for chronic airflow limitation. The St. George's Respiratory Questionnaire . Am Rev Respir Dis . 1992 ; 145 ( 6 ): 1321 – 7 . 1595997 10.1164/ajrccm/145.6.1321

[15] Jones PW , Harding G , Berry P , Wiklund I , Chen WH , Kline Leidy N. Development and first validation of the COPD Assessment Test . Eur Respir J . 2009 ; 34 ( 3 ): 648 – 54 . 19720809 10.1183/09031936.00102509

[16] Klok FA , Boon GJAM , Barco S , Endres M , Geelhoed JJM , Knauss S , et al . The Post- COVID-19 Functional Status scale: a tool to measure functional status over time after COVID-19 . European Respiratory Journal . 2020 ; 50 ( 3 ) . 10.1183/13993003.01494-2020 PMC7236834 32398306

[17] Craig CL , Marshall AL , Sjostrom M , Bauman AE , Booth ML , Ainsworth BE , et al . International physical activity questionnaire: 12-country reliability and validity . Med Sci Sports Exerc . 2003 ; 35 ( 8 ): 1381 – 95 . 12900694 10.1249/01.MSS.0000078924.61453.FB

[18] Borg GAV. Psychophysical bases of perceived exertion. Med Sci Sports Exerc. 1982;14(5):377–81. 7154893

[19] Enright PL. The Six-Minute Walk Test. Respiratory Care. 2003;48(8):783–5. 12890299

[20] Miller MR , Hankinson J , Brusasco V , Burgos F , Casaburi R , Coates A , et al . Standardisation of spirometry . Eur Respir J . 2005 ; 26 ( 2 ): 319 – 38 . 16055882 10.1183/09031936.05.00034805

[21] Graham BL , Brusasco V , Burgos F , Cooper BG , Jensen R , Kendrick A , et al . 2017 ERS/ATS standards for single-breath carbon monoxide uptake in the lung . Eur Respir J . 2017 ; 50 ( 3 ) . 10.1183/13993003.00016-2016 28049168

[22] Svenningsen S , Kirby M , Starr D , Leary D , Wheatley A , Maksym GN , et al . Hyperpolarized (3) He and (129) Xe MRI: differences in asthma before bronchodilation . J Magn Reson Imaging . 2013 ; 38 ( 6 ): 1521 – 30 . 23589465 10.1002/jmri.24111

[23] Estepar RSJ, Ross JC, Krissian K, Schultz T, Washko GR, Kindlmann GL. Computational Vascular Morphometry for the Assessment of Pulmonary Vascular Disease Based on Scale-Space Particles. 2012 9th Ieee International Symposium on Biomedical Imaging (Isbi). 2012:1479–82. 10.1109/ISBI.2012.6235851 PMC3670102 23743962

[24] Matheson A , Cunningham R , Bier E , Lu J , Driehuys B , Pickering J , et al . Hyperpolarized 129Xe pulmonary MRI and asymptomatic Atrial Septal Defect . Chest . 2022 : ( In Press ). 10.1016/j.chest.2021.11.020 35396051

[25] McFann K , Baxter BA , LaVergne SM , Stromberg S , Berry K , Tipton M , et al . Quality of Life (QoL) Is Reduced in Those with Severe COVID-19 Disease, Post-Acute Sequelae of COVID- 19, and Hospitalization in United States Adults from Northern Colorado . Int J Environ Res Public Health . 2021 ; 50 ( 3 ) . 10.3390/ijerph182111048 PMC8582735 34769566

[26] Rahaghi FN , Argemi G , Nardelli P , Dominguez-Fandos D , Arguis P , Peinado VI , et al . Pulmonary vascular density: comparison of findings on computed tomography imaging with histology . Eur Respir J . 2019 ; 50 ( 3 ) . 10.1183/13993003.00370-2019 PMC7007984 31196942

[27] Walsh CL , Tafforeau P , Wagner WL , Jafree DJ , Bellier A , Werlein C , et al . Imaging intact human organs with local resolution of cellular structures using hierarchical phase-contrast tomography . Nat Methods . 2021 . 10.1038/s41592-021-01317-x PMC8648561 34737453

[28] Mendez R , Latorre A , Gonzalez-Jimenez P , Feced L , Bouzas L , Yepez K , et al . Reduced Diffusion Capacity in COVID-19 Survivors . Ann Am Thorac Soc . 2021 ; 18 ( 7 ): 1253 – 5 . 33472019 10.1513/AnnalsATS.202011-1452RL PMC8328367

[29] Wang ZY , Rankine L , Bier EA , Mummy D , Lu JL , Church A , et al . Using hyperpolarized Xe-129 gas-exchange MRI to model the regional airspace, membrane, and capillary contributions to diffusing capacity . Journal of Applied Physiology . 2021 ; 130 ( 5 ): 1398 – 409 . 33734831 10.1152/japplphysiol.00702.2020 PMC8354826

[30] Townsend L , Dowds J , O’Brien K , Sheill G , Dyer AH , O’Kelly B , et al . Persistent Poor Health after COVID-19 Is Not Associated with Respiratory Complications or Initial Disease Severity . Ann Am Thorac Soc . 2021 ; 18 ( 6 ): 997 – 1003 . 33413026 10.1513/AnnalsATS.202009-1175OC PMC8456724

[31] Ferrer M , Villasante C , Alonso J , Sobradillo V , Gabriel R , Vilagut G , et al . Interpretation of quality of life scores from the St George's Respiratory Questionnaire . Eur Respir J . 2002 ; 19 ( 3 ): 405 – 13 . 11936515 10.1183/09031936.02.00213202

[32] Pinto LM , Gupta N , Tan W , Li PZ , Benedetti A , Jones PW , et al . Derivation of normative data for the COPD assessment test (CAT) . Respir Res . 2014 ; 15 : 68 . 24957783 10.1186/1465-9921-15-68 PMC4100027

[33] Currow DC, Plummer JL, Crockett A, Abernethy AP. A community population survey of prevalence and severity of dyspnea in adults. J Pain Symptom Manage. 2009;38(4):533–45. 19822276 10.1016/j.jpainsymman.2009.01.006

[34] Ackermann M , Verleden SE , Kuehnel M , Haverich A , Welte T , Laenger F , et al . Pulmonary Vascular Endothelialitis, Thrombosis, and Angiogenesis in Covid-19 . N Engl J Med . 2020 ; 383 ( 2 ): 120 – 8 . 32437596 10.1056/NEJMoa2015432 PMC7412750

[35] Brito-Azevedo A , Pinto EC , de Cata Preta Corrêa GA , Bouskela E. SARS-CoV-2 infection causes pulmonary shunt by vasodilatation . J Med Virol . 2021 ; 93 ( 1 ): 573 – 5 . 32706407 10.1002/jmv.26342 PMC7404894

[36] Post-COVID Conditions: Centers for Disease Control and Prevention ; 2021 [updated September 16, 2021. Available from : https://www.cdc.gov/coronavirus/2019-ncov/long-term-effects/index.html .

[37] Soriano JB , Murthy S , Marshall JC , Relan P , Diaz JV , Condition WHOCCDWGoP-C- . A clinical case definition of post-COVID-19 condition by a Delphi consensus . Lancet Infect Dis . 2021 . 10.1016/S1473-3099(21)00703-9 PMC8691845 34951953

[38] National Institute of Health and Care Excellence . COVID-19 Rapid Guideline: Managing COVID-19 . NICE ; 2021 . 34181371

[39] Stanojevic S , Graham BL , Cooper BG , Thompson BR , Carter KW , Francis RW , et al . Official ERS technical standards: Global Lung Function Initiative reference values for the carbon monoxide transfer factor for Caucasians . Eur Respir J . 2017 ; 50 ( 3 ) . 10.1183/13993003.00010-2017 28893868

